# Increased complexity of t(11;14) rearrangements in plasma cell neoplasms compared with mantle cell lymphoma

**DOI:** 10.1002/gcc.22977

**Published:** 2021-06-22

**Authors:** Joanna C. Dalland, James B. Smadbeck, Neeraj Sharma, Reid G. Meyer, Kathryn E. Pearce, Patricia T. Greipp, Jess F. Peterson, Shaji Kumar, Rhett P. Ketterling, Rebecca L. King, Linda B. Baughn

**Affiliations:** ^1^ Division of Hematopathology, Department of Laboratory Medicine and Pathology Mayo Clinic Rochester Minnesota USA; ^2^ Division of Computational Biology, Department of Quantitative Health Sciences Mayo Clinic Rochester Minnesota USA; ^3^ Division of Laboratory Genetics and Genomics, Department of Laboratory Medicine and Pathology Mayo Clinic Rochester Minnesota USA; ^4^ Division of Hematology, Department of Internal Medicine Mayo Clinic Rochester Minnesota USA

**Keywords:** *CCND1*, fluorescence in situ hybridization, *IGH*, mantle cell lymphoma, mate‐pair sequencing, next‐generation sequencing, plasma cell neoplasms, t(11;14)(q13;q32)

## Abstract

Plasma cell neoplasms (PCN) and mantle cell lymphoma (MCL) can both harbor t(11;14)(q13;q32) (*CCND1/IGH*), usually resulting in cyclin D1 overexpression. In some cases, particularly at low levels of disease, it can be morphologically challenging to distinguish between these entities in the bone marrow (BM) since PCN with t(11;14) are often CD20‐positive with lymphoplasmacytic cytology, while MCL can rarely have plasmacytic differentiation. We compared the difference in *CCND1*/*IGH* by fluorescence in situ hybridization (FISH) in PCN and MCL to evaluate for possible differentiating characteristics. We identified 326 cases of MCL with t(11;14) and 279 cases of PCN with t(11;14) from either formalin‐fixed, paraffin‐embedded tissue or fresh BM specimens. The “typical,” balanced *CCND1*/*IGH* FISH signal pattern was defined as three total *CCND1* signals, three total *IGH* signals, and two total fusion signals. Any deviation from the “typical” pattern was defined as an “atypical” pattern, which was further stratified into “gain of fusion” vs “complex” patterns. There was a significantly higher proportion of cases that showed an atypical FISH pattern in PCN compared with MCL (53% vs 27%, *P* < .0001). There was also a significantly higher proportion of cases that showed a complex FISH pattern in PCN compared with MCL (47% vs 17%, *P* < .0001). We confirmed these findings using mate‐pair sequencing of 25 PCN and MCL samples. PCN more often have a complex *CCND1*/*IGH* FISH pattern compared with MCL, suggesting possible differences in the genomic mechanisms underlying these rearrangements in plasma cells compared with B cells.

## 1 INTRODUCTION

Mantle cell lymphoma (MCL) is a mature B‐cell neoplasm most often of pre‐germinal center origin.^1^ In contrast, plasma cell neoplasms (PCN), such as plasma cell myeloma (PCM), are composed of terminally differentiated B‐cells of post‐germinal center origin which are often heavy chain class‐switched and secrete a monoclonal immunoglobulin.^1^, ^2^ Both MCL and PCN can show t(11;14)(q13;q32) (*CCND1/IGH*), occurring in >95% of cases of MCL^1^ and in ~15% of PCM.^3^ This translocation is associated with cyclin D1 overexpression, a critical regulator of the cell cycle.^4^, ^5^, ^6^ Detection of the t(11;14) in the evaluation of lymphoma is used to aid in establishing the diagnosis of MCL. In contrast, a panel of FISH probes including *CCND1*/*IGH* are evaluated in PCM for prognostic and therapeutic purposes.^3^ PCM with t(11;14) has been reported to be associated with a favorable prognosis with a median overall survival of 7 to 10 years.^3^ In addition, this translocation in the setting of PCM is associated with an increased incidence of developing plasma cell leukemia.^4^

PCN with t(11;14) often show a lymphoplasmacytic cytology and express CD20, which can make these cases especially challenging to distinguish from low‐grade B cell lymphomas with plasmacytic differentiation, which also involve the bone marrow and cause overlapping clinical features with monoclonal gammopathy of undetermined significance (MGUS) and PCM.^7^ Plasmacytic differentiation has rarely been reported in MCL cases.^8^, ^9^, ^10^, ^11^, ^12^, ^13^ While usually easily distinguished from a PCN with t(11;14) based on pathologic features, at low level infiltrates in the BM there are occasional cases which may cause diagnostic uncertainty. This differentiation can also be particularly challenging in the setting of a small biopsy with crush artifact or poor fixation. After encountering rare diagnostically challenging cases in which overlap between a low‐level MCL with plasmacytic differentiation and a true PCN with t(11;14), we sought to compare the difference in genomic patterns in PCN and MCL by fluorescence in situ hybridization (FISH) using the *CCND1*/*IGH* probe set with the goal to evaluate possible differentiating characteristics potentially to aid in diagnostically challenging cases.

## 2 MATERIALS AND METHODS

### 2.1 Case selection

This study was approved by the Mayo Clinic Institutional Review Board. The Mayo Clinic Genomics laboratory database from 2007 to 2018 was retrospectively reviewed to identify all cases of PCN and MCL investigated by FISH. Cases positive for *CCND1/IGH* were subjected to further review. Data including the final diagnosis, number of individual *CCND1* and *IGH* signals, and fusion signals were collected. Thirty‐four consult cases in which the final diagnosis was unavailable were reviewed by a hematopathologist (JCD) to confirm the pathologic diagnosis.

### 2.2 Fluorescence in situ hybridization testing

**FIGURE 1:**
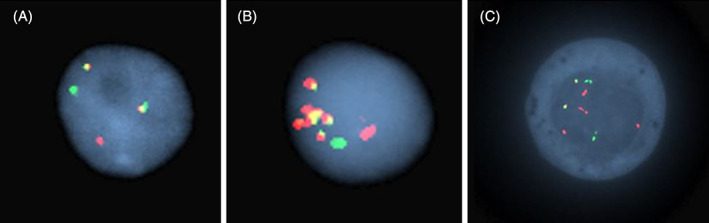
Representative FISH patterns in MCL and PCN. A, Mantle cell lymphoma case showing the “typical” balanced *CCND1*/*IGH* translocation pattern with three total *CCND1* signals (red), three total *IGH* signals (green), and two total fusion signals (yellow). B, Plasma cell myeloma showing amplification of the fusion signal (yellow). This case additionally showed a *TP53* deletion. C, Plasma cell myeloma showing atypical FISH pattern with 6 *CCND1* signals (red), 4 *IGH* signals (green) and 2 fusion signals (yellow)

Interphase FISH was performed on formalin‐fixed, paraffin‐embedded tissue (FFPE) or fresh BM specimens using standard FISH pretreatment, hybridization, and fluorescence microscopy protocols.^14^ A dual‐color, double fusion probe set for t(11;14) *CCND1/IGH*, a break‐apart probe set for *MYC* (8q24.1) rearrangement, and an enumeration probe for *TP53* deletion or monosomy 17 (centromere 17/*TP53*) were utilized (Abbott Molecular, Des Plaines, IL, for all probe sets). The *IGH* probe is labeled with Spectrum Green (Abbott Molecular) and the *CCND1* probe is labeled with Spectrum Orange (Abbott Molecular); the imaging of the Orange fluorophore is herein referred to as Red. A total of 100 or 200 interphase nuclei were evaluated in each case, with 50 or 100 nuclei evaluated independently by two qualified clinical cytogenetic technologists and interpreted by a board‐certified (American Board of Medical Genetics and Genomics) clinical cytogeneticist. The “typical,” balanced *CCND1*/*IGH* FISH signal pattern was defined as three total *CCND1* signals (red), three total *IGH* signals (green), and two total fusion signals (yellow) or 1R1G2F (Figure 1A). Any deviation from the “typical” pattern was defined as an “atypical” pattern, including both gain of fusion signals (1 or more; eg, 1R1G3F) and unbalanced/complex abnormalities (Figure 1B‐C). The percentage of cases with a “typical” pattern vs “atypical” pattern and gain of fusion vs complex pattern were compared for each group. The “atypical” patterns were further stratified into “gain of fusion” vs “complex” patterns. Groups were compared using two‐tailed Fisher's exact statistical analysis and visualized using GraphPad Prism version 8.0.0 for Windows, GraphPad Software (San Diego, CA).

### 2.3 Mate‐pair sequencing

Mate‐pair sequencing (MPseq) was performed on a subset of 16 PCN and 9 MCL cases. DNA extraction and mate‐pair library preparation methods have been previously described.^15^, ^16^, ^17^ Briefly, DNA was isolated from plasma cells as described in Smadbeck et al^18^ or from unselected MCL cells using either the Qiagen Puregene extraction kit (for samples <2 mL), Autopure LS Automated high‐quality DNA extraction (for samples >2 mL) or the QIAmp Tissue kit for fixed cell pellet samples. DNA was processed using the Illumina Nextera Mate Pair library preparation kit (Illumina, San Diego, CA) and sequenced on the Illumina HiSeq 2500 in rapid run mode as described in Smadbeck et al.^18^ Pooled libraries were hybridized onto a flow cell (two samples per lane) and sequenced using 101‐basepair reads and paired end sequencing. The sequencing data were mapped to the reference genome (GRCh38) using BIMA^19^ and analyzed using SVAtools for the detection of structural variants (SVs) (large genomic alterations (>30 Kb) that involve breakpoint junctions and/or copy number alterations (CNAs)). Detection and visualization of the breakpoint locations of junctions and CNAs utilizes the following algorithms.^15^, ^16^, ^17^

## 3 RESULTS

**FIGURE 2:**
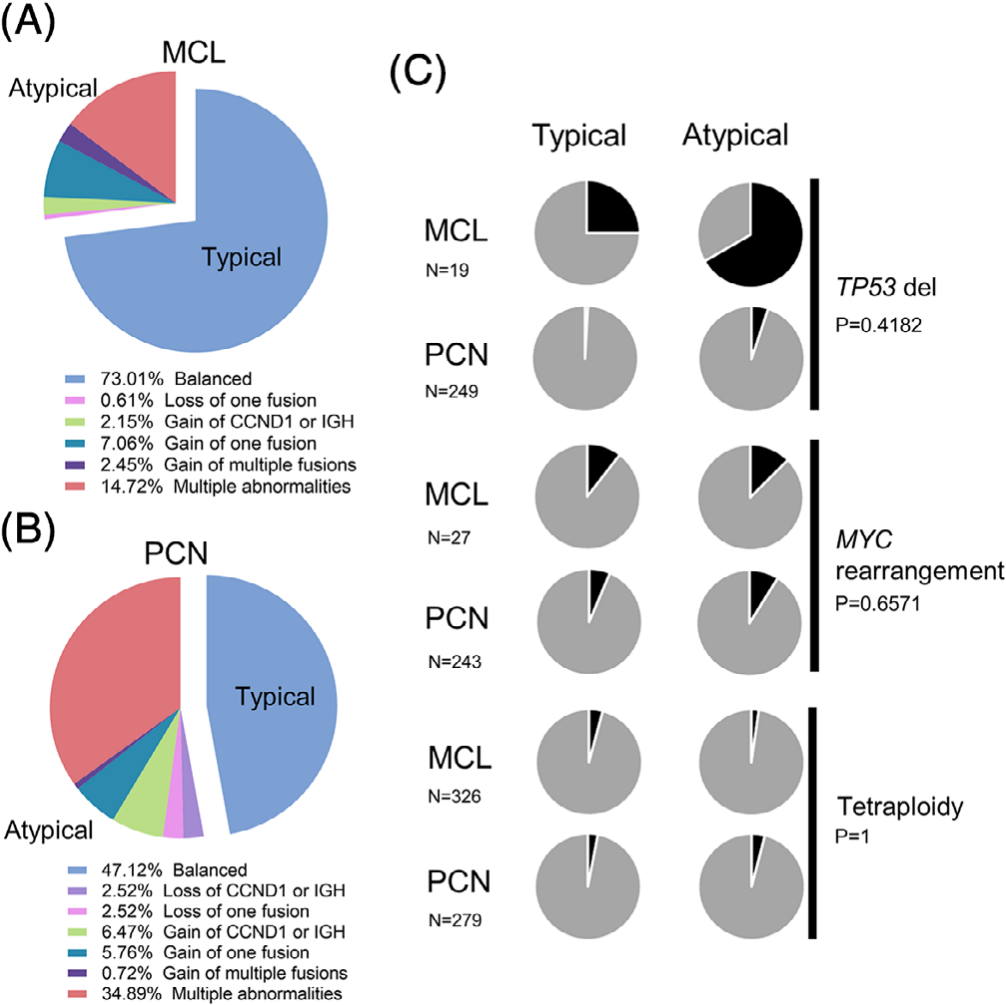
Distribution of *CCND1*/*IGH* FISH patterns observed in mantle cell lymphoma vs plasma cell neoplasms. A, Pie charts showing the fraction of MCL and PCN cases with various *CCND1*/*IGH* FISH abnormalities. B, Pie charts showing the fraction of cases with *TP53* deletions, *MYC* rearrangements, and tetraploidy (black shading) in typical and atypical MCL and PCN cases

To determine possible differentiating characteristics of *CCND1*/*IGH* FISH complexity between MCL and PCM, we analyzed the *CCND1*/*IGH* FISH patterns for 326 cases of MCL with *CCND1*/*IGH* (275 FFPE specimens; 51 BM specimens) and 279 cases of PCN with *CCND1*/*IGH* (56 FFPE specimens; 223 BM specimens). A variety of FISH patterns were observed, including balanced translocations, the gain of fusion, loss of fusion, and a combination of abnormalities (Figure 1, Figure 2A,B). In both FFPE and BM specimens, there was a significantly higher proportion of cases that showed an atypical *CCND1*/*IGH* FISH pattern in PCN compared with MCL (53% vs 27%, *P* < .0001) (Table 1). We further divided the atypical category into those with a simple gain of *CCND1*/*IGH* fusion signal, and complex representing any other atypical FISH result. There was a significantly higher proportion of cases that showed a complex FISH pattern in PCN compared with MCL (47% vs 17%, *P* < .0001) (Table 1). One PCN FFPE specimen showed amplification of the fusion signal (Figure 1B). These data demonstrate that cases of PCN were nearly 2 to 3‐fold more likely to have an atypical or complex *CCND1*/*IGH* FISH result compared with MCL.

**TABLE 1:** Distribution of *CCND1*/*IGH* FISH Patterns

			Atypical pattern (unbalanced)			
		Typical pattern (balanced)	Total atypical cases	Gain of fusion	Complex	Total cases
MCL	FFPE	197 (72%)	78 (28%)	25 (9%)	53 (19%)	275
	BM	41 (80%)	10 (20%)	6 (12%)	4 (8%)	51
	Total	238 (73%)	88 (27%)	31 (10%)	57 (17%)	326
PCN	FFPE	21 (38%)	35 (63%)	3 (5%)	32 (57%)	56
	BM	110 (49%)	113 (51%)	15 (7%)	98 (44%)	223
	Total	131 (47%)	148 (53%)	18 (6%)	130 (47%)	279

*Note*: Typical balanced 1R1G2F t(11;14) FISH pattern and any abnormal t(11;14) FISH pattern that deviates from 1R1G2F is considered atypical unbalanced. Percentages in parenthesis refer to the total number of cases in the full cohort.

Abbreviations: BM, bone marrow; FFPE, formalin fixed paraffin embedded; FISH, fluorescence in situ hybridization; MCL, mantle cell lymphoma; PCN, plasma cell neoplasm.

We evaluated whether *CCND1*/*IGH* FISH complexity was associated with an increased incidence of *TP53* deletion, *MYC* rearrangement, or tetraploidy.*TP53* deletion is associated with increased genomic complexity and poorer outcome in both PCN and MCL, including an association with the more highly proliferative blastoid and pleomorphic variants of MCL.^1^, ^3^, ^20^ A *TP53* deletion was identified in six cases (32%) of MCL and eight cases (3.2%) of PCN (Table 2A, Figure 2C). The distribution of *TP53* deletion among the *CCND1*/*IGH* FISH subtypes (typical vs atypical) was not considered significant (*P* = .4182). *MYC* rearrangements can also occur as a secondary cytogenetic abnormality and contribute to progression in PCN^1^, ^3^, ^21^, ^22^ and are associated with a higher proliferation in MCL.^20^ A *MYC* rearrangement was identified in 3 cases of MCL (11%) and 19 cases of PCN (7.8%) (Table 2B, Figure 2C). Similar to *TP53* deletion, the distribution of *MYC* rearrangement among the *CCND1*/*IGH* FISH subtypes (typical vs atypical) was also not considered significant (*P* = .6571). A tetraploid clone, reported to be more common in pleomorphic and blastoid variants of MCL,^23^ was seen in 12 cases of MCL (3.7%) and 10 cases of PCN (3.6%) with a similar distribution among the *CCND1*/*IGH* FISH subtypes (typical vs atypical) (*P* = 1) (Table 2C, Figure 2C).

**TABLE 2:** Cases with concurrent *TP53*, *MYC*, and tetraploidy

A	*TP53* deletion with *CCND1*/*IGH* typical pattern	*TP53* deletion with *CCND1*/*IGH* atypical pattern	Total cases with *TP53* evaluation
MCL	4 (21.1%, 25.0%)	2 (10.5%, 66.7%)	19 (16 typical/3 atypical)
PCN	1 (0.4%, 0.9%)	7 (2.8%, 5.1%)	249 (112 typical/137 atypical)

B	*MYC* rearrangement with *CCND1*/*IGH* typical pattern	*MYC* rearrangement with *CCND1*/*IGH* atypical pattern	Total cases with *MYC* evaluation
MCL	2 (7.4%, 10.5%)	1 (3.7%, 12.5%)	27 (19 typical/8 atypical)
PCN	7 (2.9%, 6.4%)	12 (4.9%, 9.0%)	243 (109 typical/134 atypical)

C	Tetraploidy with *CCND1*/*IGH* typical pattern	Tetraploidy with *CCND1*/*IGH* atypical pattern	Total cases with tetraploid evaluation
MCL	10 (3.1%, 4.2%)	2 (0.6%, 2.3%)	326 (238 typical/88 atypical)
PCN	4 (1.4%, 3.1%)	6 (2.2%, 4.1%)	279 (131 typical/148 atypical)

*Note*: First percentage reflects fraction of abnormal cases over total cases evaluated and second percentage reflects fraction of abnormal cases over total typical or atypical cases evaluated.

Abbreviations: MCL, mantle cell lymphoma; PCN, plasma cell neoplasm.

**FIGURE 3:**
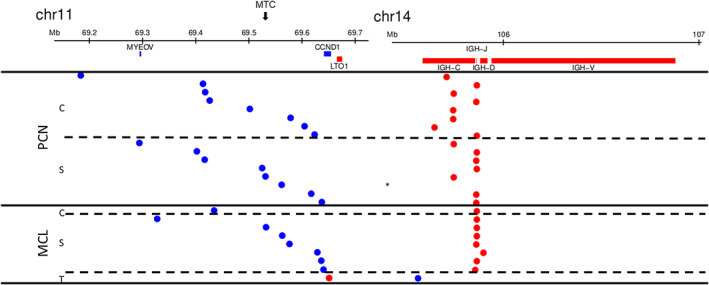
Breakpoint locations for t(11;14) translocations. Breakpoint locations in chromosomes 11 and 14 for the t(11;14) translocations are depicted in relation to the *CCND1* and *IGH*‐C/J/D/V. Each translocation is shown as two dots in the same row. A blue dot indicates that the translocation occurs on the forward strand, a red on the reverse strand. The translocations are split by disease (PCN and MCL) and by whether the translocation belongs to a complex rearrangement (C), a simple rearrangement (S), or is a *CCND1* 3′ UTR truncation event similar to that reported in Nadeu et al^20^ and Menke et al^5^ (T). A star indicates a case where the t(11;14) translocation is part of a rearrangement where the *CCND1* region does not connect directly to the *IGH* locus by a single junction. Rather, it connects to the *ZFYVE21* gene. There is a 124 Kb gain of the *IGH* constant region and insertion of this portion within the *KLC1* gene 38 Kb centromeric to the *ZFYVE21* gene. Major translocation cluster (MTC) of t(11;14) in MCL is indicated

**FIGURE 4:**
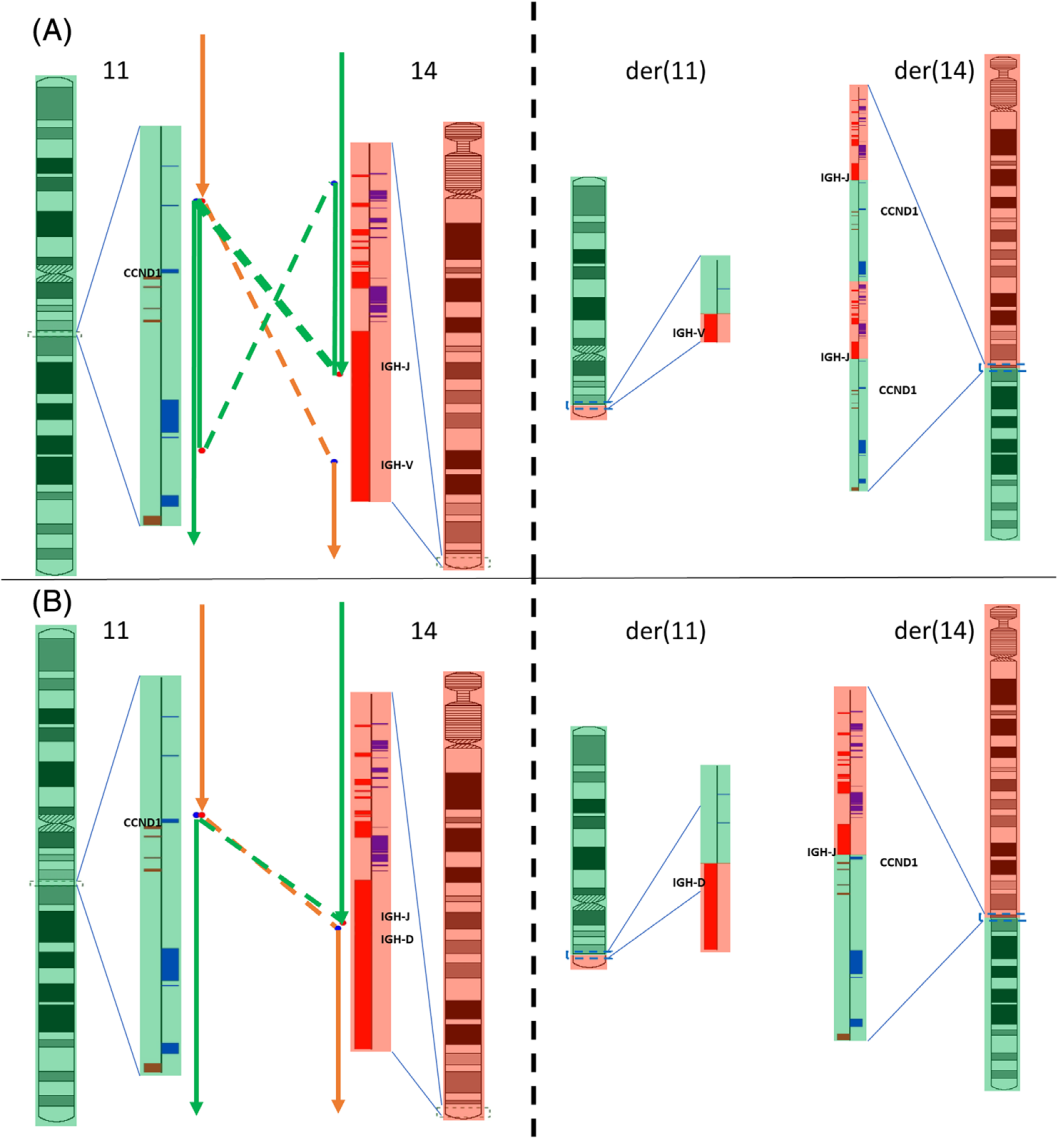
Complete reconstruction of a complex and simple t(11;14) translocation. A, Reconstruction of a complex t(11;14) translocation (P1) and B, reconstruction of a simple t(11;14) translocation (M1). The left side of each panel is each event as depicted against the reference genome. The right side of each panel is each event as they exist in the der (11)/der (14) chromosomes. Regions that are derived from chromosome 11 are depicted in light green and regions that are derived from chromosome 14 are depicted in light red. Zoomed in regions of the chromosomes show *IGH* and *CCND1* in relation to the rearrangement. Other genes in the regions are unlabeled and depicted as blue and red boxes if they are on the forward or reverse strand of the chromosomes, respectively. Orange and green lines show the path of reconstruction for the der (11) and der (14) chromosomes, respectively. Dashed lines denote junctions that connect discordant regions of the genome. Double lines denote regions or junctions that are passed through twice in the reconstruction and thus indicate regions/junctions that have been gained and are depicted twice in the derivative chromosomes. If no lines overlap a region that indicates an area of loss and are not depicted in the derivative chromosomes

MPseq has been shown to be superior to FISH in characterizing rearrangement complexity.^15^ We evaluated the t(11;14) rearrangement in a subset of MCL and PCN cases by MPseq. The analysis included nine cases of MCL for which we had fresh sample available and 16 cases of PCN previously described in Smadbeck et al.^18^ MPseq typically provides junction information within 1 Kb of the breakpoint. As expected, analysis of the *CCND1* breakpoint locations revealed that the breakpoints of the t(11;14) translocation in MCL were located in a region closer to the *CCND1* gene (chr11:69 641 156‐69 654 474, GRCh38) compared with PCN (Table 3, Figure 3). Analysis of the *IGH* breakpoint locations revealed that the breakpoints in MCL were exclusively found in the VDJ region of *IGH*, while 8/16 breakpoints in PCN were located in the *IGH* constant region (Table 3, Figure 3). In addition, 3/9 MCL samples were found more frequently to harbor an 11q deletion telomeric to *CCND1* ^24^ compared with only 1/16 PCN cases. The 11q deletions across the four cases ranged from 10.3 to 29.4 Mb in size with a shared region of 1.6 Mb (chr11:107 406 532‐108 999 346, GRCh38) which includes the *ATM* and *DDX10* genes. In addition, eight samples of PCN had evidence of a gain near *CCND1* which would result in a gain of the *CCND1*/*IGH* fusion (Figures 4A and Figure S1A‐H). The gains were typically <1 Mb in size and included the region around the *CCND1*/*IGH* fusion on chromosomes 11 and 14. These samples constituted 8/9 of the complex cases sequenced, demonstrating that a large majority of the complex cases sequenced were complex because of this phenotypic structural change. This type of complexity was absent in all nine MCL samples analyzed by MPseq, which were more likely to be simple, balanced rearrangements as depicted in Figure 4B. Similar to our findings by FISH analysis, our MPseq data also show that PCN demonstrates a more complex t(11;14) pattern in comparison to MCL.

**TABLE 3:** Cases analyzed by MPseq

Case	FISH ISCN	Class	Orientation	Breakpoint *CCND1*	Breakpoint *IGH*	Fusion	*IGH* Loc
P1	nuc ish(MYCx2)(5′MYC sep 3′MYCx1),(CCND1‐XT,IGH‐XT)x3(CCND1‐XT con IGH‐XTx2)/(CCND1‐XT,IGH‐XT)x4(CCND1‐XT con IGH‐XTx3),(RB1,LAMP1)x1,(TP53x1,D17Z1x2)	Complex	T	69 413 951	105 863 347	M	J
			C	69 413 776	106 422 207		V
P2	nuc ish(TP73x2,1q22x3),(CCND1‐XT,IGH‐XT)x4‐5(CCND1‐XT con IGH‐XTx3‐4)/(CCND1‐XTx1,CCND1‐XT amp,IGH‐XTx1,IGH‐XT amp)(CCND1‐XT amp con IGH‐XT amp),(RB1,LAMP1)x1	Complex	T	69 604 906	105 647 460	M	IGHG2‐S
			C	69 604 906	105 647 460		IGHG2‐S
P3	nuc ish(TP73x2,1q22x5),(D3Z1,D9Z1,D15Z4)x3,(CCND1‐XT,IGH‐XT)x3(CCND1‐XT con IGH‐XTx2),(RB1,LAMP1)x1	Simple	T	69 294 297	105 746 023	S	IGHG1‐S
			C	69 294 297	105 746 023		IGHG1‐S
P4	nuc ish(TP73x1,1q22x3–4)/(TP73x2,1q22x6),(D3Z1,D7Z1,D9Z1,D15Z4)x4,(5′MYCx3,3′MYCx2)(5′MYC con 3′MYCx2)/(5′MYCx6,3′MYCx4)(5′MYC con 3′MYCx4),(CCND1‐XT,IGH‐XT)x3(CCND1‐XT con IGHx2)/(CCND1‐XT,IGH‐XT)x4(CCND1‐XT con IGHx3)/(CCND1‐XT,IGH‐XT)x5(CCND1‐XT con IGHx4),(TP53x1,D17Z1x2)	Complex	T	69 418 042	105 746 066	M	IGHG1‐S
			C	69 418 042	105 746 066		IGHG1‐S
P5	nuc ish(TP73x2,1q22x3),(CCND1‐XT,IGH‐XT)x3(CCND1‐XT con IGH‐XTx2)	Simple	T	69 531 419	105 746 307	S	IGHG1‐S
			C	69 531 419	105 746 307		IGHG1‐S
P6	nuc ish(CCND1‐XTx3,IGH‐XTx2)(CCND1‐XT con IGHx2)/(CCND1‐XTx5,IGH‐XTx4)(CCND1‐XT con IGHx4),(TP53x1,D17Z1x2)	Complex	T	69 425 842	105 862 578	M	J
			C	69 425 842	105 862 578		J
P7	nuc ish(CCND1‐XT,IGH‐XT)x4(CCND1‐XT con IGH‐XTx3),(TP53x1,D17Z1x2)	Complex	T	69 623 547	105 863 783	M	J
			C	69 623 318	106 116 754		V
P8	nuc ish(TP73,1q22,MYC,RB1,LAMP1,TP53,D17Z1)x4,(D3Z1,D7Z1,D9Z1,D15Z4)x3‐4,(CCND1‐XTx6,IGH‐XTx7)(CCND1‐XT con IGH‐XTx4)	Simple	T	69 561 305	103 718 610	S	IGHA1‐S*
			C	69 561 075	103 719 240		N/A
P9	nuc ish(CCND1‐XT,IGH‐XT)x4(CCND1‐XT con IGH‐XTx3),(RB1x1,LAMP1x2)	Complex	T	69 502 329	105 744 360	M	IGHG1‐S
			C	69 502 329	105 744 360		IGHG1‐S
P10	nuc ish(CCND1‐XTx3,IGH‐XTx2)(CCND1‐XT con IGH‐XTx1)	Complex	T	69 577 992	105 745 163	M	IGHG1‐S
P11	nuc ish(CCND1‐XTx3),(IGH‐XTx3),(CCND1‐XT con IGH‐XTx2)	Simple	T	69 416 622	105 862 149	S	J
			C	69 416 622	105 862 149		J
P12	nuc ish(CCND1‐XTx3,IGH‐XTx2)(CCND1‐XT con IGH‐XTx1),(RB1,LAMP1)x1	Simple	T	69 617 673	105 861 717	S	J
P13	nuc ish(CCND1‐XTx2),(IGH‐XTx2),(CCND1‐XT con IGH‐XTx1)/(CCND1‐XTx2),(IGH‐XTx3),(CCND1‐XT con IGH‐XTx1)	Simple	T	69 636 797	105 861 502	S	J
P14	nuc ish(TP73x2,1q22x4),(D3Z1x3–4),(D7Z1,D9Z1,D15Z4,TP53,D17Z1)x3,(5′MYCx4,3′MYCx2)(5′MYC con 3′MYCx2)/(5′MYCx6,3′MYCx3)(5′MYC con 3′MYCx3),(CCND1‐XT,IGH‐XT)x6(CCND1‐XT con IGH‐XTx5)	Complex	T	69 183 682	105 711 033	M	IGHA1‐S
			C	69 183 682	105 711 033		IGHA1‐S
P15	nuc ish(MYC,RB1,LAMP1)x1,(CCND1‐XT,IGH‐XT)x3(CCND1‐XT con IGH‐XTx2)	Simple	T	69 524 666	105 864 080	S	J
			C	69 524 666	106 211 500		V
P16	nuc ish(TP73x2,1q22x3),(5′MYCx2,3′MYCx1)(5′MYC con 3′MYCx1),(CCND1‐XT,IGH‐XT)x3(CCND1‐XT con IGH‐XTx2)	Simple	T	69 402 179	105 864 520	S	J
			C	69 401 157	105 881 545		D
							
M1	nuc ish(CCND1‐XTx3),(IGH‐XTx3),(CCND1‐XT con IGH‐XTx2)[434/500]	Simple	T	69 629 361	105 898 085	S	D
			C	69 629 361	105 898 085		D
M2	nuc ish(CCND1‐XTx3),(IGHx3),(CCND1‐XT con IGHx2)[458/500]	Simple	T	69 640 225	105 857 384	S	C‐J
			C	69 640 179	105 904 708		D
M3	nuc ish(CCND1‐XTx3),(IGHx3),(CCND1‐XT con IGHx2)[430/500]	Simple	T	69 531 968	105 864 286	S	J
			C	69 531 968	105 864 286		J
M4	nuc ish(CCND1‐XTx3),(IGHx3),(CCND1‐XT con IGHx2)[284/500]	Simple	T	69 562 218	105 863 830	S	J
			C	69 561 405	106 593 440		V
M5	nuc ish(CCND1‐XTx3),(IGH‐XTx3),(CCND1‐XT con IGH‐XTx2)[254/500]	Simple	T	69 575 981	105 861 663	S	C‐J
			C	69 574 683	105 913 227		D
M6	nuc ish(CCND1‐XTx3),(IGHx3),(CCND1‐XT con IGHx2)[120/500]/(CCND1‐XTx4),(IGHx4),(CCND1‐XT con IGHx3)[234/500]	Complex	T	69 435 003	105 864 155	M	J
			C	69 434 668	105 916 887		D
M7	nuc ish(CCND1‐XTx3),(IGHx3),(CCND1‐XT con IGHx2)[120/500]/(CCND1‐XTx4),(IGHx4),(CCND1‐XT con IGHx3)[234/500]	Simple	C	69 651 119	105 563 982	TR	N/A
M8	nuc ish(CCND1‐XTx3,IGH‐XTx2)(CCND1‐XT con IGH‐XTx1)[235/500]	Simple	T	69 635 967	105 864 016	S	J
M9	nuc ish(CCND1‐XTx4),(IGH‐XTx3),(CCND1‐XT con IGH‐XTx2)[428/500]	Simple	T	69 327 612	105 865 603	S	J
			C	69 327 612	105 865 603		J

Abbreviations: C, centromeric; ISCN, international system for human cytogenetic nomenclature; M, mantle cell lymphoma; M, multiplied; P, plasma cell neoplasm; S, single; T, telomeric; TR, truncated.

## 4 DISCUSSION

We show that PCNs have a significantly higher propensity to have atypical and complex *CCND1*/*IGH* FISH patterns compared with MCL. This increased complexity in t(11;14) suggests differences in the genomic mechanisms underlying these rearrangements in PCs compared with B cells. The breakpoint in *CCND1* typically occurs at the 5′ end (centromeric) in both PCN and MCL, with an increased frequency of breakpoints clustered near the major translocation cluster in MCL. However, the *IGH* breakpoint has been shown to be different in MCL vs PCN.^25^, ^26^ In MCL, the majority of breaks occur in the VDJ region of *IGH* and frequently involves the IGHV3‐23 and IGHV4‐59 genes.^25^, ^27^, ^28^ In contrast, the breakpoints identified in PCN are variable and are usually located in an *IGH* switch region.^6^, ^29^, ^30^ Numerous variants have been identified in PCN, including different 11q13 breakpoints, deletions of variable and constant *IGH* segments, duplications and losses of the *IGH* gene on the normal non‐translocated chromosome 14 as well as *IGH*/*CCND1* fusion on der (14) and *CCND1*/*IGH* fusions on der (11).^26^, ^31^, ^32^

Previous studies showed that *IGH* rearrangements in MCL appeared to be due to aberrant VDJ recombination (RAG1/2 mediated), while in PCN *IGH* rearrangements appeared to be due to aberrant class switch recombination (AID mediated).^29^, ^30^, ^33^ However, there is evidence that AID can also cooperate with RAG to mediate t(11;14) in MCL, as evidenced by breaks near AID hotspots.^28^ Open and active chromatin structure could allow AID accessibility to mediate the DNA breaks and subsequent translocation.^20^, ^28^ The gains of *CCND1* and *IGH* observed in PCN cases could be described as templated insertions, a previously reported complex event found in about 20% of plasma cell myeloma cases.^34^ In contrast, templated insertions do not appear to be a common feature in MCL.^20^ The reason for these differences in the incidence of templated insertions between PCN and MCL requires additional investigation.

The single nucleotide polymorphism rs603965 (also known as rs9344) occurring at the splice site of *cyclin D1* leading to the 870G > A polymorphism has been reported to be associated with a risk of t(11;14) PCM and AL amyloidosis.^35^, ^36^ In contrast, the rs603965 genotype showed no relationship with MCL risk.^35^, ^37^ These findings most likely reflect the different underlying mechanisms associated with the development of t(11;14) in PCN vs MCL. We also evaluated whether the complex *CCND1/IGH* positive cases were more likely to have *TP53* deletions or *MYC* rearrangements; however, our sample size was small and, our findings were not considered significant. We also investigated the presence of tetraploid clones; however, there was also no significant difference in the association of tetraploidy with the complex *CCND1*/*IGH* positive cases. In our cohort, only one PCN case with t(11;14) amplification was identified, while no MCL cases with amplification were observed. It is noted that previous studies have shown that amplification can also be seen in MCL,^20^, ^38^, ^39^ and the lack of MCL cases with amplification in our cohort may represent sampling bias. In addition, increased complexity has been previously reported to be associated with the blastoid variant of MCL^38^; however, we were unable to assess for this association in our cohort due to the absence of pathology reports for all cases. Limitations of this study include the small sample size of cases evaluated for *TP53* deletions and *MYC* rearrangements and the absence of clinical outcome for all cases, limiting evaluation of the prognostic significance of FISH complexity in PCN and MCL. Similar to our FISH results, our MPseq data also show that PCN demonstrates a more complex t(11;14) pattern in comparison to MCL. However, the sample size tested by MPseq was also small. At the time of this study, we did not have the ability to perform MPseq on FFPE samples and required fresh tissue for testing, thus limiting MCL case selection for MPseq.

Our studies indicate that PCNs have a significantly increased frequency of atypical and complex *CCND1*/*IGH* FISH patterns compared with MCL. While we initially sought to determine if this could potentially be used to aid in the diagnosis of challenging cases, despite the significant difference we observed, there remains significant overlap with a subset of MCL cases associated with a complex FISH pattern, thus limiting the application of these findings for diagnostic purposes. Future studies to evaluate whether there is any association of genomic complexity to clinical outcome may be useful to determine if this observation may be of prognostic significance.

## CONFLICT OF INTERESTS

Shaji Kumar served as a consultant for Celgene, Takeda, Amgen, Janssen, and Bristol‐Myers Squibb and received research funding from Celgene, Takeda, Novartis, Amgen, AbbVie, Janssen, and Bristol‐Myers Squibb. The remaining authors declare no competing financial interests.

## Supporting information

**SUPPLEMENTARY FIGURE 1** Reconstruction of complex t(11;14) translocation in PCN. Partial reconstruction of 8 samples of PCN that had a gain near *CCND1* resulting in gain of the *IGH/CCND1* fusion. P1 in A, P2 in B, P4 in C, P6 in D, P7 in E, P9 in F, P10 in G and P14 in H. Due to the complexity and existence of subclonal variation in the rearrangement in patient 2, what is depicted is one possible solution to the rearrangement. The actual rearrangement is more complex and varied in structure. In patient 4, both sides of the t(11;14) balanced junction have evidence of complexity. What is depicted is the portion of the complex rearrangement which includes two copies of the CCND1 gene and two copies of the *IGH*/CCND1 fusion. Due to the complexity and existence of subclonal variation in the rearrangement in patient 6, what is depicted is one possible solution to the rearrangement. The actual rearrangement is more complex and varied in structure. In patient 9, due to the telomeric nature of the chr2 templated insertion, the connection between chr2 and chr22 is extrapolated from copy number changes. For this reason, this is a plausible rearrangement, however possibly a simplified version of the actual rearrangement. It is possible that the actual rearrangement is more complex than depicted. The complexity in patient 10 is subclonal H. Like the other complexities this rearrangement in patient 14 results in a copy of the t(11;14). However, unlike the others, this does not result in a copy of *CCND1*. Instead the complexity jumps to the *MYC* region of chr8 and would bring *IGH* in proximity to both *MYC* and *CCND1* in the course of the complex rearrangement. Zoomed in regions of the chromosomes show IGH and *CCND1* in relation to the rearrangement. Other genes in the regions are unlabeled and depicted as blue and red boxes if they are on the forward or reverse strand of the chromosomes, respectively. Green lines show the path of reconstruction. Dashed lines denote junctions that connect discordant regions of the genome. Double lines denote regions or junctions that are passed through twice in the reconstruction and thus indicate regions/junctions that have been gained and are depicted twice in the derivative chromosomes. If no lines overlap a region, this indicates an area of loss and these regions are not depicted in the derivative chromosomes

## Data Availability

The data that support the findings of this study are available from the corresponding author upon reasonable request.
