# Supplementary figures and images for: Increased complexity of t(11;14) rearrangements in plasma cell neoplasms compared with mantle cell lymphoma
Source: Genes Chromosomes Cancer. 2021 Jun 22;60(10):678–86. doi: 10.1002/gcc.22977 (PMC8453742; doi:10.1002/gcc.22977)

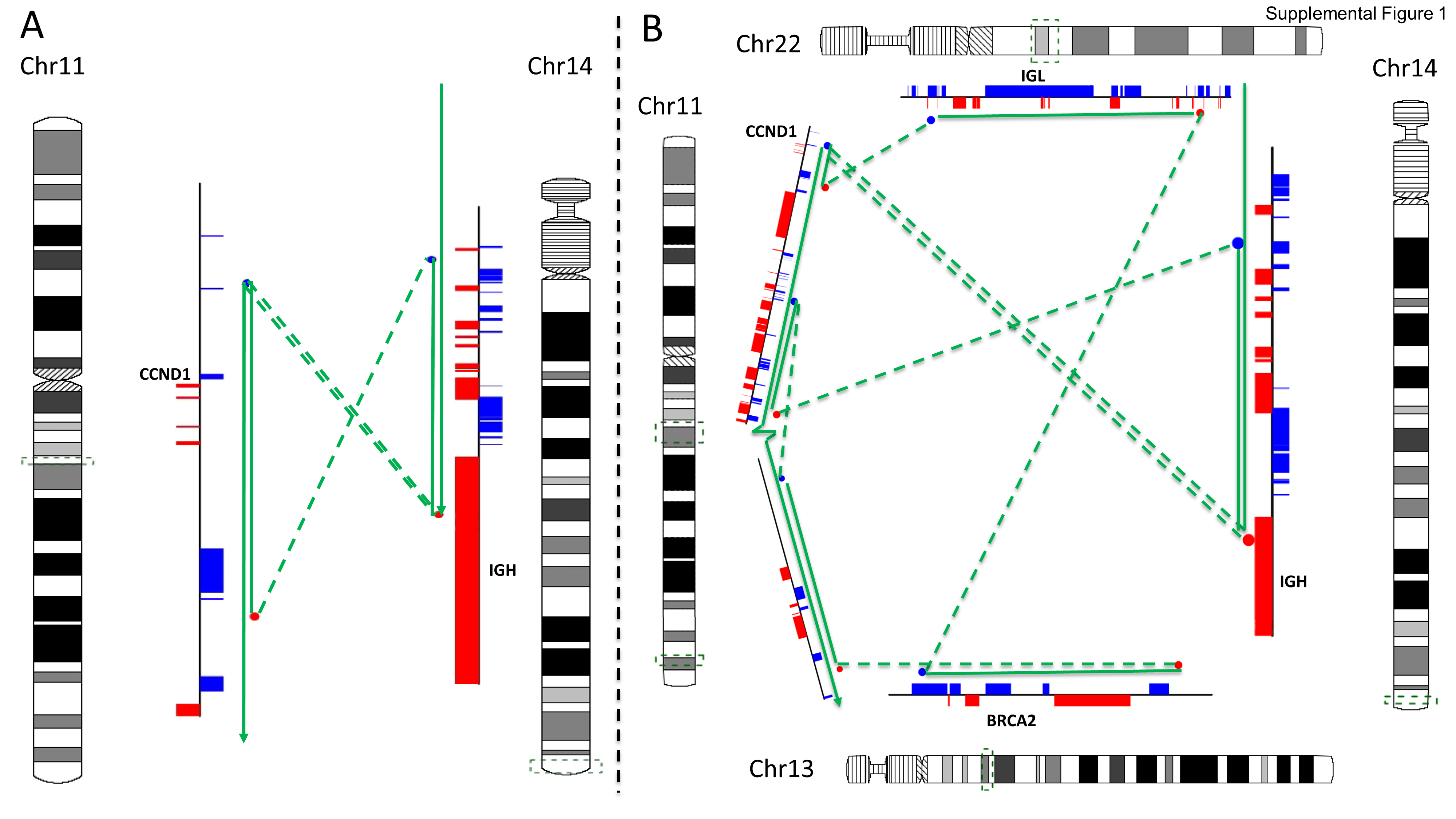

Supplement: Supplementary file 1 — SUPPLEMENTARY FIGURE 1 Reconstruction of complex t(11;14) translocation in PCN. Partial reconstruction of 8 samples of PCN that had a gain near CCND1 resulting in gain of the IGH/CCND1 fusion. P1 in A, P2 in B, P4 in C, P6 in D, P7 in E, P9 in F, P10 in G and P14 in H. Due to the complexity and existence of subclonal variation in the rearrangement in patient 2, what is depicted is one possible solution to the rearrangement. The actual rearrangement is more complex and varied in structure. In patient 4, both sides of the t(11;14) balanced junction have evidence of complexity. What is depicted is the portion of the complex rearrangement which includes two copies of the CCND1 gene and two copies of the IGH/CCND1 fusion. Due to the complexity and existence of subclonal variation in the rearrangement in patient 6, what is depicted is one possible solution to the rearrangement. The actual rearrangement is more complex and varied in structure. In patient 9, due to the telomeric nature of the chr2 templated insertion, the connection between chr2 and chr22 is extrapolated from copy number changes. For this reason, this is a plausible rearrangement, however possibly a simplified version of the actual rearrangement. It is possible that the actual rearrangement is more complex than depicted. The complexity in patient 10 is subclonal H. Like the other complexities this rearrangement in patient 14 results in a copy of the t(11;14). However, unlike the others, this does not result in a copy of CCND1. Instead the complexity jumps to the MYC region of chr8 and would bring IGH in proximity to both MYC and CCND1 in the course of the complex rearrangement. Zoomed in regions of the chromosomes show IGH and CCND1 in relation to the rearrangement. Other genes in the regions are unlabeled and depicted as blue and red boxes if they are on the forward or reverse strand of the chromosomes, respectively. Green lines show the path of reconstruction. Dashed lines denote junctions that connect di [file GCC-60-678-s001.zip › GCC_22977_Slide5.TIF]

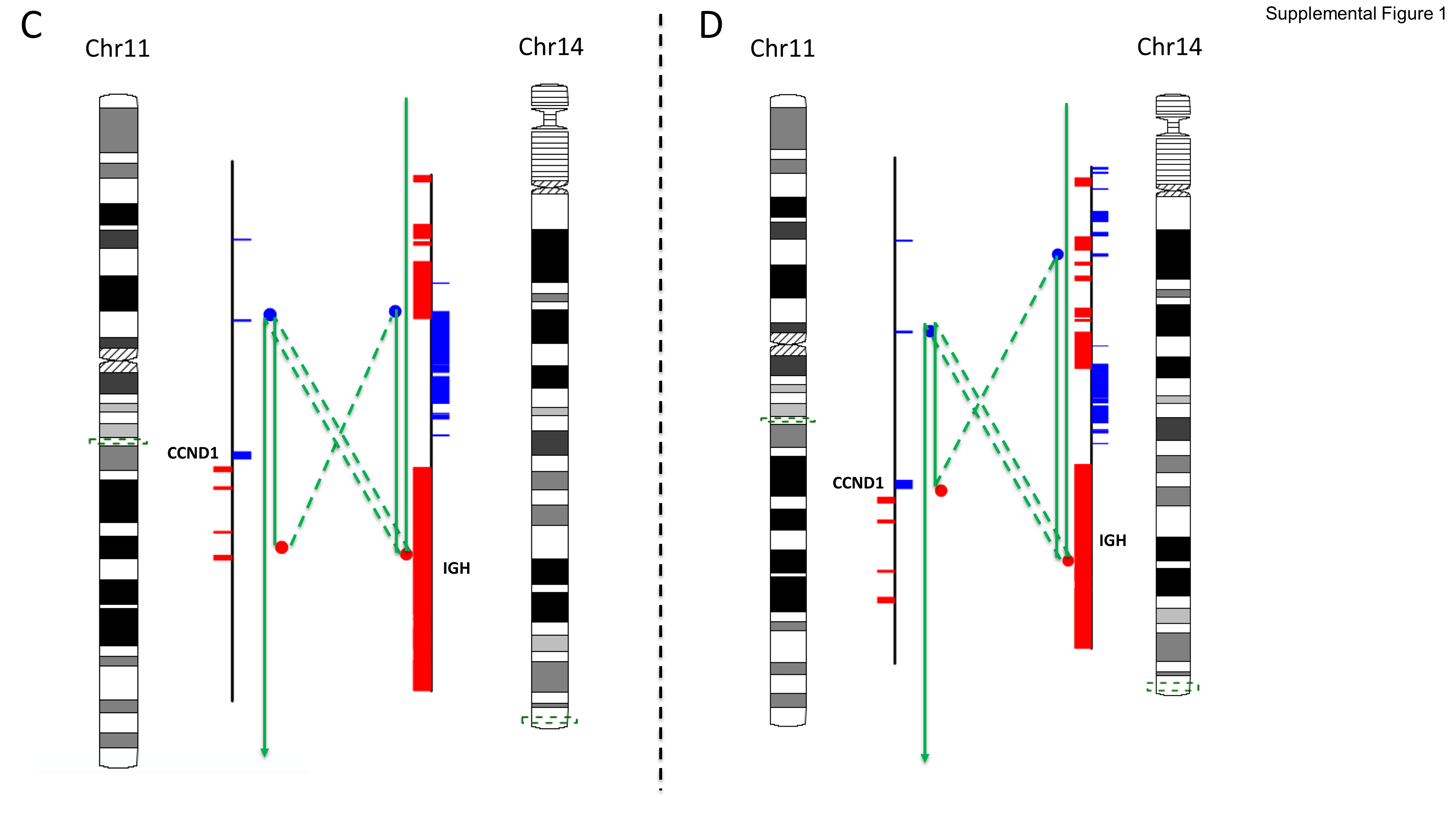

Supplement: Supplementary file 1 — SUPPLEMENTARY FIGURE 1 Reconstruction of complex t(11;14) translocation in PCN. Partial reconstruction of 8 samples of PCN that had a gain near CCND1 resulting in gain of the IGH/CCND1 fusion. P1 in A, P2 in B, P4 in C, P6 in D, P7 in E, P9 in F, P10 in G and P14 in H. Due to the complexity and existence of subclonal variation in the rearrangement in patient 2, what is depicted is one possible solution to the rearrangement. The actual rearrangement is more complex and varied in structure. In patient 4, both sides of the t(11;14) balanced junction have evidence of complexity. What is depicted is the portion of the complex rearrangement which includes two copies of the CCND1 gene and two copies of the IGH/CCND1 fusion. Due to the complexity and existence of subclonal variation in the rearrangement in patient 6, what is depicted is one possible solution to the rearrangement. The actual rearrangement is more complex and varied in structure. In patient 9, due to the telomeric nature of the chr2 templated insertion, the connection between chr2 and chr22 is extrapolated from copy number changes. For this reason, this is a plausible rearrangement, however possibly a simplified version of the actual rearrangement. It is possible that the actual rearrangement is more complex than depicted. The complexity in patient 10 is subclonal H. Like the other complexities this rearrangement in patient 14 results in a copy of the t(11;14). However, unlike the others, this does not result in a copy of CCND1. Instead the complexity jumps to the MYC region of chr8 and would bring IGH in proximity to both MYC and CCND1 in the course of the complex rearrangement. Zoomed in regions of the chromosomes show IGH and CCND1 in relation to the rearrangement. Other genes in the regions are unlabeled and depicted as blue and red boxes if they are on the forward or reverse strand of the chromosomes, respectively. Green lines show the path of reconstruction. Dashed lines denote junctions that connect di [file GCC-60-678-s001.zip › GCC_22977_Slide6.TIF]

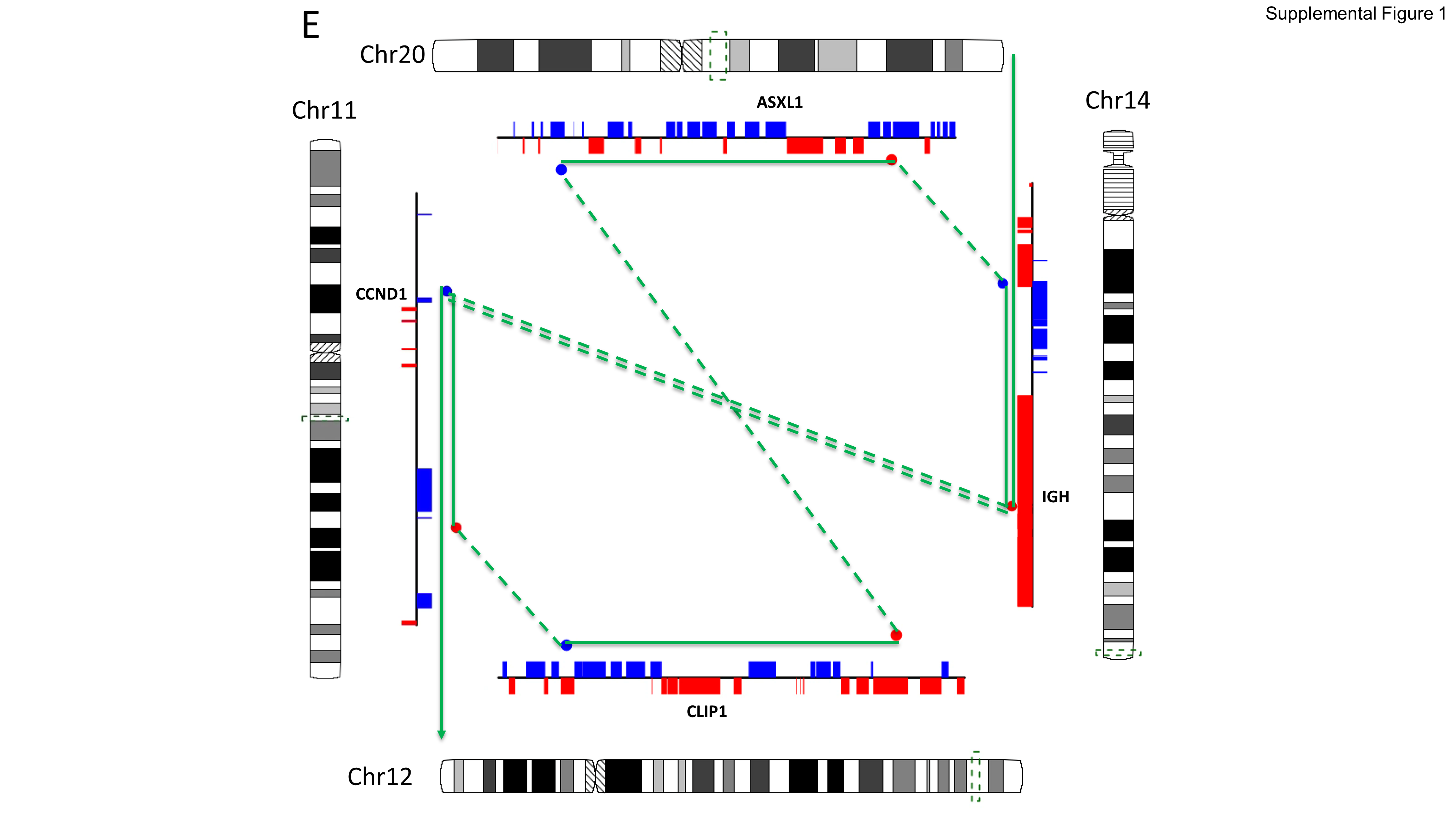

Supplement: Supplementary file 1 — SUPPLEMENTARY FIGURE 1 Reconstruction of complex t(11;14) translocation in PCN. Partial reconstruction of 8 samples of PCN that had a gain near CCND1 resulting in gain of the IGH/CCND1 fusion. P1 in A, P2 in B, P4 in C, P6 in D, P7 in E, P9 in F, P10 in G and P14 in H. Due to the complexity and existence of subclonal variation in the rearrangement in patient 2, what is depicted is one possible solution to the rearrangement. The actual rearrangement is more complex and varied in structure. In patient 4, both sides of the t(11;14) balanced junction have evidence of complexity. What is depicted is the portion of the complex rearrangement which includes two copies of the CCND1 gene and two copies of the IGH/CCND1 fusion. Due to the complexity and existence of subclonal variation in the rearrangement in patient 6, what is depicted is one possible solution to the rearrangement. The actual rearrangement is more complex and varied in structure. In patient 9, due to the telomeric nature of the chr2 templated insertion, the connection between chr2 and chr22 is extrapolated from copy number changes. For this reason, this is a plausible rearrangement, however possibly a simplified version of the actual rearrangement. It is possible that the actual rearrangement is more complex than depicted. The complexity in patient 10 is subclonal H. Like the other complexities this rearrangement in patient 14 results in a copy of the t(11;14). However, unlike the others, this does not result in a copy of CCND1. Instead the complexity jumps to the MYC region of chr8 and would bring IGH in proximity to both MYC and CCND1 in the course of the complex rearrangement. Zoomed in regions of the chromosomes show IGH and CCND1 in relation to the rearrangement. Other genes in the regions are unlabeled and depicted as blue and red boxes if they are on the forward or reverse strand of the chromosomes, respectively. Green lines show the path of reconstruction. Dashed lines denote junctions that connect di [file GCC-60-678-s001.zip › GCC_22977_Slide7.TIF]

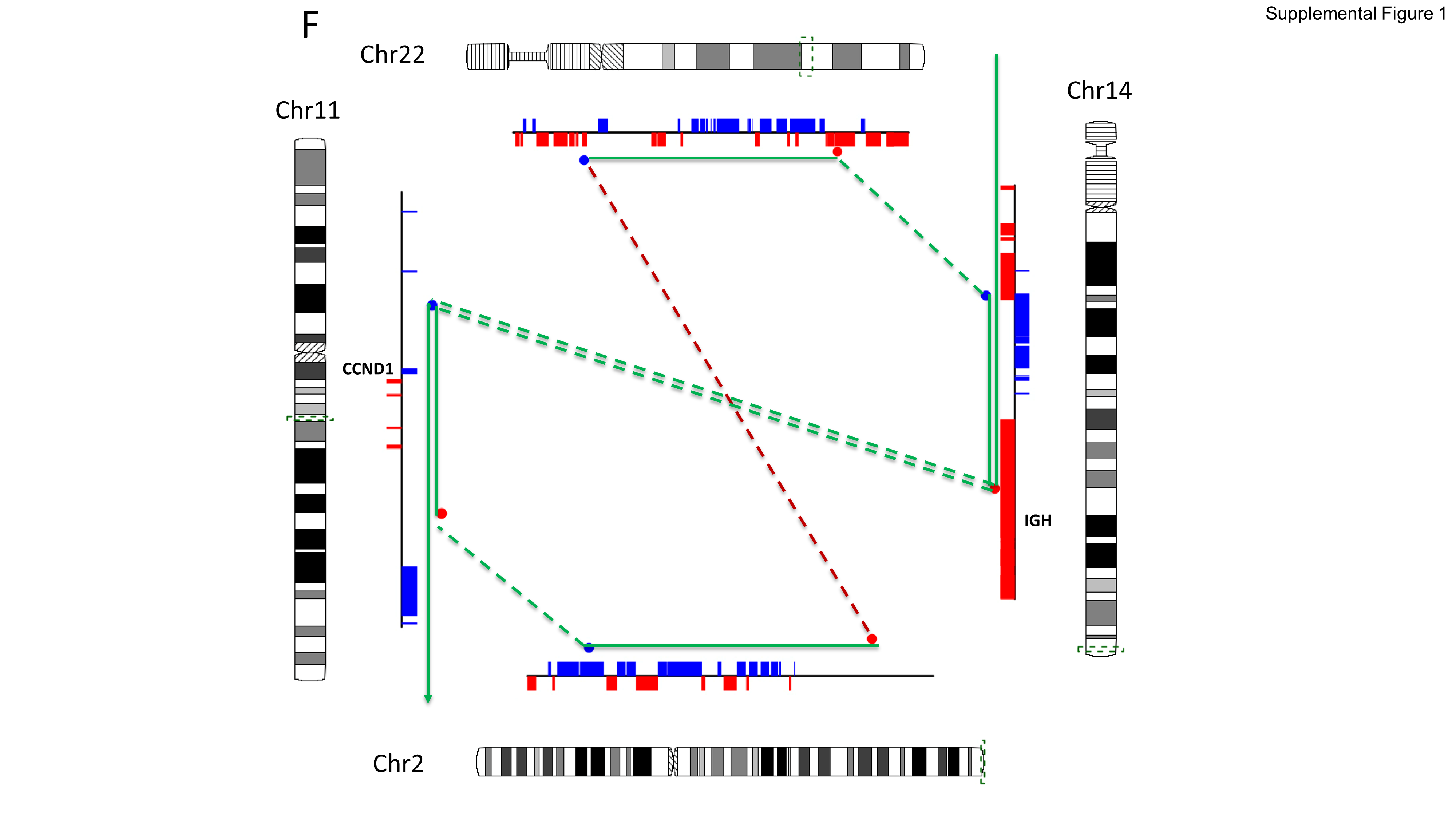

Supplement: Supplementary file 1 — SUPPLEMENTARY FIGURE 1 Reconstruction of complex t(11;14) translocation in PCN. Partial reconstruction of 8 samples of PCN that had a gain near CCND1 resulting in gain of the IGH/CCND1 fusion. P1 in A, P2 in B, P4 in C, P6 in D, P7 in E, P9 in F, P10 in G and P14 in H. Due to the complexity and existence of subclonal variation in the rearrangement in patient 2, what is depicted is one possible solution to the rearrangement. The actual rearrangement is more complex and varied in structure. In patient 4, both sides of the t(11;14) balanced junction have evidence of complexity. What is depicted is the portion of the complex rearrangement which includes two copies of the CCND1 gene and two copies of the IGH/CCND1 fusion. Due to the complexity and existence of subclonal variation in the rearrangement in patient 6, what is depicted is one possible solution to the rearrangement. The actual rearrangement is more complex and varied in structure. In patient 9, due to the telomeric nature of the chr2 templated insertion, the connection between chr2 and chr22 is extrapolated from copy number changes. For this reason, this is a plausible rearrangement, however possibly a simplified version of the actual rearrangement. It is possible that the actual rearrangement is more complex than depicted. The complexity in patient 10 is subclonal H. Like the other complexities this rearrangement in patient 14 results in a copy of the t(11;14). However, unlike the others, this does not result in a copy of CCND1. Instead the complexity jumps to the MYC region of chr8 and would bring IGH in proximity to both MYC and CCND1 in the course of the complex rearrangement. Zoomed in regions of the chromosomes show IGH and CCND1 in relation to the rearrangement. Other genes in the regions are unlabeled and depicted as blue and red boxes if they are on the forward or reverse strand of the chromosomes, respectively. Green lines show the path of reconstruction. Dashed lines denote junctions that connect di [file GCC-60-678-s001.zip › GCC_22977_Slide8.TIF]

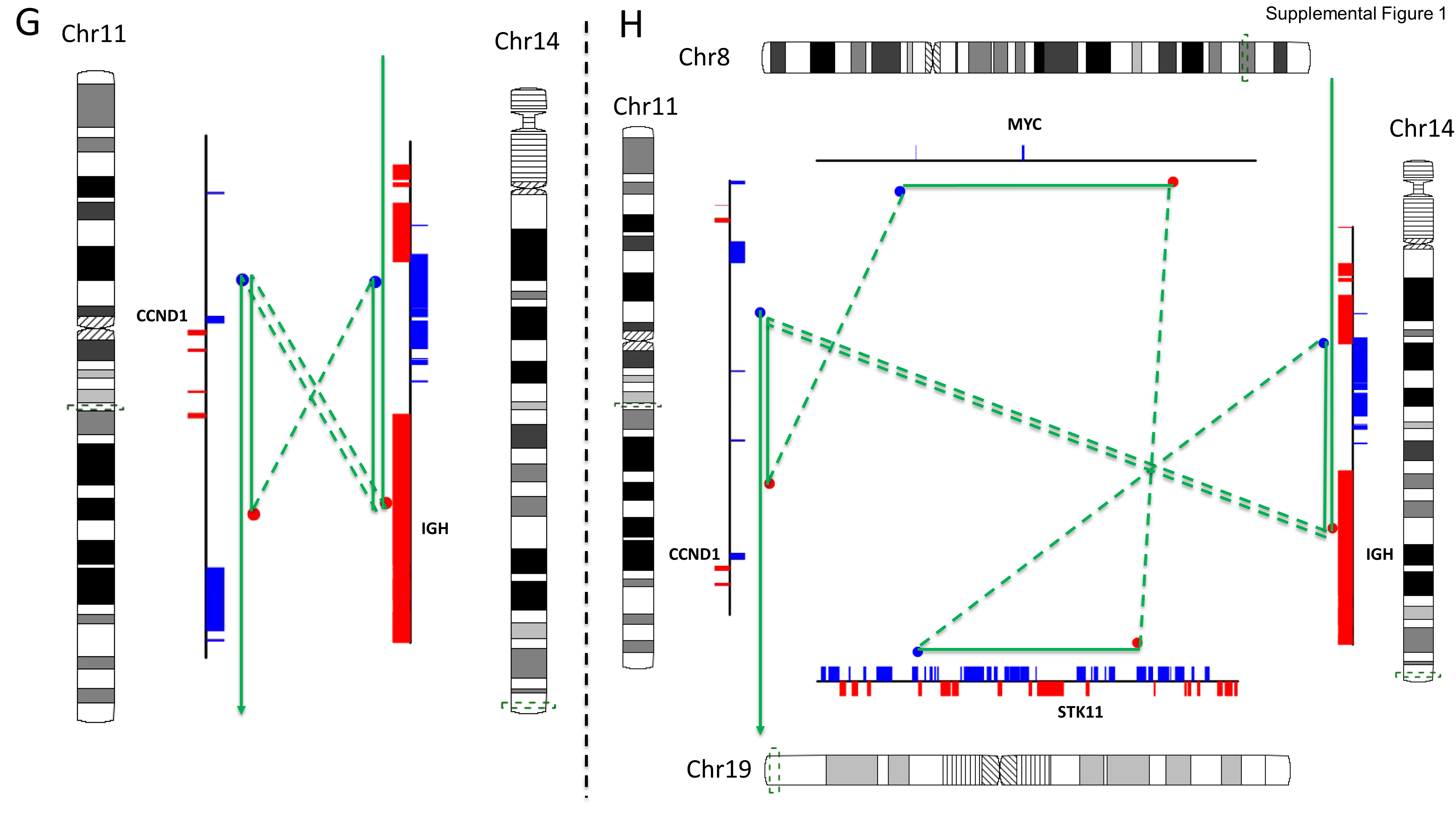

Supplement: Supplementary file 1 — SUPPLEMENTARY FIGURE 1 Reconstruction of complex t(11;14) translocation in PCN. Partial reconstruction of 8 samples of PCN that had a gain near CCND1 resulting in gain of the IGH/CCND1 fusion. P1 in A, P2 in B, P4 in C, P6 in D, P7 in E, P9 in F, P10 in G and P14 in H. Due to the complexity and existence of subclonal variation in the rearrangement in patient 2, what is depicted is one possible solution to the rearrangement. The actual rearrangement is more complex and varied in structure. In patient 4, both sides of the t(11;14) balanced junction have evidence of complexity. What is depicted is the portion of the complex rearrangement which includes two copies of the CCND1 gene and two copies of the IGH/CCND1 fusion. Due to the complexity and existence of subclonal variation in the rearrangement in patient 6, what is depicted is one possible solution to the rearrangement. The actual rearrangement is more complex and varied in structure. In patient 9, due to the telomeric nature of the chr2 templated insertion, the connection between chr2 and chr22 is extrapolated from copy number changes. For this reason, this is a plausible rearrangement, however possibly a simplified version of the actual rearrangement. It is possible that the actual rearrangement is more complex than depicted. The complexity in patient 10 is subclonal H. Like the other complexities this rearrangement in patient 14 results in a copy of the t(11;14). However, unlike the others, this does not result in a copy of CCND1. Instead the complexity jumps to the MYC region of chr8 and would bring IGH in proximity to both MYC and CCND1 in the course of the complex rearrangement. Zoomed in regions of the chromosomes show IGH and CCND1 in relation to the rearrangement. Other genes in the regions are unlabeled and depicted as blue and red boxes if they are on the forward or reverse strand of the chromosomes, respectively. Green lines show the path of reconstruction. Dashed lines denote junctions that connect di [file GCC-60-678-s001.zip › GCC_22977_Slide9.TIF]
